# Achievable rate maximization for decode-and-forward MIMO–OFDM networks with an energy harvesting relay

**DOI:** 10.1186/s40064-016-2203-8

**Published:** 2016-05-17

**Authors:** Guanyao Du, Jianjun Yu

**Affiliations:** Computer Network Information Center, Chinese Academy of Sciences, Beijing, 100190 China

**Keywords:** Energy harvesting (EH), Simultaneous wireless information and power transfer (SWIPT), MIMO–OFDM, Decode-and-forward (DF), Augmented Lagrangian penalty function (ALPF)

## Abstract

This paper investigates the system achievable rate for the multiple-input multiple-output orthogonal frequency division multiplexing (MIMO–OFDM) system with an energy harvesting (EH) relay. Firstly we propose two protocols, time switching-based decode-and-forward relaying (TSDFR) and a flexible power splitting-based DF relaying (PSDFR) protocol by considering two practical receiver architectures, to enable the simultaneous information processing and energy harvesting at the relay. In PSDFR protocol, we introduce a temporal parameter to describe the time division pattern between the two phases which makes the protocol more flexible and general. In order to explore the system performance limit, we discuss the system achievable rate theoretically and formulate two optimization problems for the proposed protocols to maximize the system achievable rate. Since the problems are non-convex and difficult to solve, we first analyze them theoretically and get some explicit results, then design an augmented Lagrangian penalty function (ALPF) based algorithm for them. Numerical results are provided to validate the accuracy of our analytical results and the effectiveness of the proposed ALPF algorithm. It is shown that, PSDFR outperforms TSDFR to achieve higher achievable rate in such a MIMO–OFDM relaying system. Besides, we also investigate the impacts of the relay location, the number of antennas and the number of subcarriers on the system performance. Specifically, it is shown that, the relay position greatly affects the system performance of both protocols, and relatively worse achievable rate is achieved when the relay is placed in the middle of the source and the destination. This is different from the MIMO–OFDM DF relaying system without EH. Moreover, the optimal factor which indicates the time division pattern between the two phases in the PSDFR protocol is always above 0.8, which means that, the common division of the total transmission time into two equal phases in previous work applying PS-based receiver is not optimal.

## Background

### Introduction

The orthogonal frequency division multiplexing (OFDM) is a key transmission technology to provide higher spectral efficiency, which has been adopted in various standards, e.g., IEEE 802.11n and 3GPP-Long Term Evolution (LTE). Moreover, the multiple-input multiple-output (MIMO) system has been extensively studied due to its ability to collect spatial diversity and provide higher system capacity (Loa et al. 2010). The combination of MIMO and OFDM technologies is believed to be further able to improve the system performance.

Recently, efforts have been made to apply MIMO and OFDM technologies together to the next-generation wireless communication systems in order to support high data rates and provide high spectral efficiency (Loa et al. 2010; Guvensen and Yilmaz 2013). The next-generation wireless communication systems are expected to support multiple users and to guarantee the quality of service (QoS). However, the performance may be limited by the available energy in the mobile devices for some practical application scenarios. For example, in wireless sensor networks (WSN) or wireless body area networks (WBAN), the nodes are usually powered by batteries which have limited lifetime. Once the power is exhausted, the batteries need to be replaced or recharged (Abreu et al. 2014).

An alternative solution to this problem is to harvest energy from the surrounding environment, which is referred to as energy harvesting (EH) technique. Conventional EH harvests energy from solar, wind, thermoelectric effects or other physical phenomena (Raghunathan et al. 2006). However, these natural energy sources may not be suitable for mobile devices and not be available in indoor environments. Recently, one prospective way is to harvest energy from the ambient radio-frequency (RF) signals (Varshney 2008), which is also referred to as simultaneous wireless information and power transfer (SWIPT).

The idea of SWIPT was first proposed in (Varshney 2008), which is based on the fact that RF signals can carry both energy and information at the same time. Primary works about SWIPT considered the ideal receiver which was assumed to be able to decode information and extract energy from the RF signal at the same time (Grover and Sahai 2010; Xiang and Tao 2012; Fouladgar and Simeone 2012). Later, this assumption was proved to be hard to realize in practical systems due to the fact that the information and energy receivers in practice operate with very different power sensitivity (i.e., −10 dBm for energy receivers and −60 dBm for information receivers) (Liu et al. 2013). And the authors in (Zhou et al. 2013) proposed two practically realizable receiver architectures, namely, time switching (TS) and power splitting (PS) architectures. These two practical architectures were further applied to point-to-point communications system (Popovski et al. 2013), two-way relaying system (Du et al. 2015) or other complex communication systems, e.g. MIMO (Zhang and Ho 2013; Shi et al. 2014; Zhou et al. 2015) and OFDM systems (Zhou et al. 2014; Ng et al. 2013a,b; Xiong et al. 2014; Di et al. 2014).

As is known, the relaying technology is an important and effective technology in wireless networks to achieve coverage extension, spectral efficiency improvement and energy saving (Loa et al. 2010). Thus, much attention has been paid to the combination of relaying technology and MIMO–OFDM (Chalise et al. 2012, 2013; Xiong et al. 2015). However, to the best of our knowledge, only a few works can be found investigating the SWIPT based on the practical realizable receiver architectures in the OFDM relaying system or MIMO relaying system or MIMO–OFDM relaying system, and even fewer of them considered SWIPT in a MIMO–OFDM relaying systems where all the nodes in networks are deployed with multiple antennas. Although existing research work has been conducted to investigate the SWIPT for the MIMO–OFDM systems, these schemes cannot be directly applied to the MIMO–OFDM systems with the assistance of a relay.

## Related work

Primary works studied the system performance of SWIPT in the end-to-end systems (Zhang and Ho 2013; Shi et al. 2014; Zhou et al. 2014; Ng et al. 2013a, b; Xiong et al. 2014). Specifically, in (Zhang and Ho 2013), a three node MIMO broadcasting system was considered, where the rate-energy bound and region were studied. In (Shi et al. 2014), the SWIPT in a multi-user multiple-input single-output (MISO) downlink system was investigated, where the total transmission power at BS was minimized by jointly designing transmit beamforming vectors and received PS ratios for all mobile stations. In (Zhou et al. 2014), a multi-user OFDM system was studied by considering both TS and PS receivers, and the optimal design of SWIPT was obtained. In (Ng et al. 2013a) and (Ng et al. 2013b), an OFDM downlink communication system with multiple mobile receivers was considered, where a resource allocation algorithm was designed to maximize the energy efficiency of the data transmission. In (Xiong et al. 2014), a resource allocation strategy was proposed to optimize the tradeoff between the downlink and uplink’s energy efficiency (EE) in a time-division duplexing (TDD) OFDM network with one access point (AP) and multiple users, where the PS receiver was deployed at each user.

Later, much attention has been paid to the combination of relaying technology and MIMO or the combination of relaying technology and OFDM (Zhou et al. 2015; Di et al. 2014). Specifically, in (Zhou et al. 2015), the system achievable rate was maximized by jointly optimized the antenna selection and power splitting in a three node two-hop system, where the EH relay deployed multiple antennas. In (Di et al. 2014), the system achievable rate was analyzed and optimized in a two-hop OFDM system, where a DF relay was employed.

Recently, some researches focused on the system performance of SWIPT in the MIMO–OFDM relaying system which employed amplify-and-forward (AF) cooperation scheme (Chalise et al. 2012, 2013; Xiong et al. 2015). Specifically, in (Chalise et al. 2012) and (Chalise et al. 2013), the optimum performance boundaries and the rate-energy region were investigated in a two-hop MIMO–OFDM system where the destination was composed of one information receiver and one energy receiver and the source and relay were assumed to be two energy-supplied nodes. In (Xiong et al. 2015), SWIPT was studied in a two-hop non-regenerative MIMO–OFDM relaying networks, where the system achievable rates were analyzed and optimized.

To the best of our knowledge, there have been no researches involving the SWIPT in a two-hop DF relaying system. As is known, as one of two basic relaying protocols, i.e., AF relaying and DF relaying, it makes sense to investigate the performance of DF relaying protocol in a MIMO–OFDM relaying system. Although some existing work can be found in the MIMO–OFDM DF end-to-end systems, these schemes cannot be directly applied to the MIMO–OFDM systems with the assistance of a relay, which is due to the reason that the SWIPT technique brings a new degree of freedom for the MIMO–OFDM system’s design. It thus motivates our investigation of the SWIPT in the MIMO–OFDM DF relaying systems.

The work most similar to ours is (Xiong et al. 2015). Compared with the work in (Xiong et al. 2015), some differences of our work are deserved to be stressed as follows. Firstly, in (Xiong et al. 2015), the author analyzed the achievable rates for the MIMO–OFDM AF relaying systems. To be noted that the analytical methods for AF and DF relaying systems are totally different. Specifically, in AF systems, the performances mainly depend on the end-to-end SNR, so the key point is to derive the end-to-end SNR, whereas in DF systems, the achievable rate of each subchannel is limited by the minimal rate over the two hops, which makes the way of deriving the end-to-end SNR no longer works for DF systems. Secondly, in (Xiong et al. 2015), the author adopted a simple equal time division scheme in PS-based protocol, which was like most previous work applying PS-based receiver, whereas in our work, we introduce a temporal parameter to describe the time division pattern between the two phases in the proposed PSDFR protocol, and design algorithms to obtain the optimal temporal parameter. Compared with the equal time division for the PS-based protocol in (Xiong et al. 2015), our protocol is more flexible and more general. Numerical results show that, the common division of the total transmission time into two equal phases in previous work is not optimal. Thirdly, compared with the work in (Xiong et al. 2015), we design different methods to solve the achievable rate maximization problems, and propose an augmented Lagrangian penalty function (ALPF) based algorithm which is independent and adaptive to solve nonlinear optimization problems with constraints (due to the reason that various methods can be used to solve the sub-problems in ALPF). At last, comparing the system performance with that in (Xiong et al. 2015) through numerical results, we find some performance differences between the AF system and the DF system, and get some important conclusions.

### Contributions

In this paper, we focus on the SWIPT for a two-hop MIMO–OFDM DF relaying system, where a source transmits its information to the destination with the help of an energy-constrained relay. The relay can harvest energy from the RF signals that it received from the source, and uses all the harvested energy to relay the information for the destination.

The main contributions of this paper can be summarized as follows:

- Firstly, we propose two protocols, time switching-based decode-and-forward relaying (TSDFR) and a flexible power splitting-based DF relaying (PSDFR) protocol by considering two practical receiver architectures, to enable the simultaneous information processing and energy harvesting at the relay. Specifically, most of the existing investigations involving PS scheme adopt equal time divisions of transmission phases, whereas in our proposed PSDFR protocol, we introduce a new temporal parameter to describe the time division pattern between the two phases in the PSDFR protocol. Compared with the equal time division of the two phases of PSR protocol in (Xiong et al. 2015), our protocol is more flexible and more general.
- Secondly, to evaluate the system performance, we discuss the system achievable rate theoretically for the two proposed protocols, respectively.
- In order to explore the system performance limit, we formulate two optimization problems for the proposed protocols to maximize the system achievable rate. Since the problems are non-convex and difficult to solve, we first analyze them theoretically and get some explicit results, then design an ALPF based algorithm which is a independent and adaptive method to solve nonlinear optimization problems with constraints.
- Finally, numerical results are provided to demonstrate the accuracy of the analytical results and the effectiveness of the proposed ALPF method. It is shown that, PSDFR outperforms TSDFR to achieve higher achievable rate in such a MIMO–OFDM relaying system. Moreover, some important conclusions are obtained: The optimal factor which indicates the time division pattern between the two phases in the PSDFR protocol is always above 0.8, which means that, the common division of the total transmission time into two equal phases in previous work applying PS-based receiver is not optimal. It is also shown that the relay position greatly affects the system performance of both protocols, and relatively worse achievable rates are achieved when the relay is placed in the middle of the source and the destination. This is different from the MIMO–OFDM DF relaying system without SWIPT. Besides, we find some differences between AF and DF systems. For example, in the AF system of Reference (Xiong et al. 2015), as the relay node moves from the source to the destination, the optimal time assignment factor in TS-based protocol will decrease to achieve higher system performance. But in DF systems, as the relay node moves from the source to the destination, the optimal time assignment factor first increases and then decreases, which is to say that, when the relay is in the middle of the source and the destination, the optimal time assignment factor is relatively high.

## Methods

### Assumptions and notations

We consider a half-duplex two-hop relaying system which consists of a source S, a destination D, and an energy-constrained relay R. All nodes are equipped with multiple antennas, and the number of antennas at S, R and D are denoted by $N_{\text{S}}$, $N_{\text{R}}$ and $N_{\text{D}}$, respectively.

S has fixed energy supplying and wants to transmit information to D. Due to the great attenuation caused by long distance or barriers between S and D, the direct link S–D is unavailable. Thus, R is used to assist the information forwarding from S to D. The energy constrained R relies on external charging since it has no internal energy source. Specifically, R harvests energy from the received RF signals transmitted from S, and uses all the harvested energy to assist the information relaying. In practical systems, R can be either idle mobile users (in cellular network) or sleeping nodes (in wireless sensor network) which are battery driven and lack of energy-supply. We assume that R operates in a half-duplex mode and all nodes have perfect knowledge of the channels of both hops.

Broadband communication OFDM is considered in this model, and the frequency-selective channel with total frequency band $\varvec{B}$ is divided into *K* frequency-flat sub-channels. Moreover, we consider the block fading channel, where the channel gain of each sub-channels remains constant during each round of relaying transmission.

### Basic process of transmission in MIMO–OFDM DF system

In this subsection, we shall describe the basic process of transmission in such a MIMO–OFDM DF relaying system, the received signal and the achievable rate are also analyzed which will be used in the following sections.

In the source phase, ${\text{S}}$ delivers the signal vector $\varvec{s}_{k} \in$ ℂ $^{{N_{\text{S}} \times 1}}$ to ${\text{R}}$, and the received signal at ${\text{R}}$ over the $k{\text{th}}$ subcarrier can be represented as

1 $${\mathbf{y}}_{{{\text{R}},k}} ={\mathbf{W}}_{{{\text{R}},k}} {\mathbf{H}}_{1,k} {\mathbf{F}}_{{{\text{S}},k}} \varvec{s}_{k} + {\mathbf{n}}_{{{\text{R}},k}},$$

where ${\text{E}}[\varvec{s}_{k} \varvec{s}_{k}^{H}] = {\mathbf{I}}_{{N_{\text{S}}}}$, ${\mathbf{H}}_{1,k} \in {\mathbb{C}}^{{N_{\text{R}} \times N_{\text{S}}}}$ denotes the channel matrix from ${\text{S}}$ to ${\text{R}}$ at hop-1 over the $k{\text{th}}$ subcarrier, and ${\mathbf{F}}_{{{\text{S}},k}} \in{\mathbb{C}}^{{N_{\text{S}} \times N_{\text{S}}}}$ denotes the precoding matrix at S. ${\mathbf{n}}_{{{\text{R}},k}} \sim CN(0,\sigma_{\text{R}}^{2} {\mathbf{I}}_{{N_{\text{R}}}})$ is a $N_{\text{R}} \times 1$ additive white Gaussian noise (AWGN) vector at ${\text{R}}$, and ${\mathbf{W}}_{{{\text{R}},k}} \in$ ℂ $^{{N_{\text{R}} \times N_{\text{R}}}}$ is the receiver filter deployed at ${\text{R}}$. Then, the achievable rate at ${\text{R}}$ is given by

2 $${\mathbf{R}}_{{{\text{R}},k}} ={ \log }_{2} \left| {{\mathbf{I}} + {\mathbf{W}}_{{{\text{R}},k}} {\mathbf{H}}_{1,k} {\mathbf{F}}_{{{\text{S}},k}} {\mathbf{F}}_{{{\text{S}},k}}^{H} {\mathbf{H}}_{1,k}^{H} {\mathbf{W}}_{{{\text{R}},k}}^{H} \sigma_{\text{R}}^{- 2}} \right|.$$

In the relay phase, ${\text{R}}$ decodes the signal $\varvec{s}_{k}$ from (1) and forwards it to ${\text{D}}$ by multiplying a forwarding matrix ${\mathbf{F}}_{{{\text{R}},m}} \in$ ℂ $^{{N_{\text{R}} \times N_{\text{R}}}}$. If the $k{\text{th}}$ subcarrier over hop-1 in the source phase is paired with the $m{\text{th}}$ subcarrier over hop-2 in the relay phase, we call them subcarrier pair (SP) $(k,m)$, and the received signal at ${\text{D}}$ over the SP $(k,m)$ can be expressed as

3 $${\mathbf{y}}_{{{\text{D}},m}} ={\mathbf{W}}_{{{\text{D}},m}} {\mathbf{H}}_{2,m} {\mathbf{F}}_{{{\text{R}},m}} \varvec{s}_{k} + {\mathbf{n}}_{{{\text{D}},m}},$$

where ${\mathbf{H}}_{2,m} \in$ ℂ $^{{N_{\text{D}} \times N_{\text{R}}}}$ denotes the channel matrix from ${\text{R}}$ to ${\text{D}}$ at hop 2 over the $m{\text{th}}$ subcarrier, and ${\mathbf{W}}_{{{\text{D}},m}} \in$ ℂ $^{{N_{\text{D}} \times N_{\text{D}}}}$ is the receiver filter deployed at ${\text{D}}$. ${\mathbf{n}}_{{{\text{D}},m}} \sim CN(0,\sigma_{\text{D}}^{2} {\mathbf{I}}_{{N_{\text{D}}}})$ is the $N_{\text{D}} \times 1$ AWGN vector at ${\text{D}}$. Then, the achievable rate at ${\text{D}}$ is given by

4 $${\mathbf{R}}_{{{\text{D}},m}} = { \log }_{2} \left| {{\mathbf{I}} + {\mathbf{W}}_{{{\text{D}},m}} {\mathbf{H}}_{2,m} {\mathbf{F}}_{{{\text{R}},m}} {\mathbf{F}}_{{{\text{R}},m}}^{H} {\mathbf{H}}_{2,m}^{H} {\mathbf{W}}_{{{\text{D}},m}}^{H} \sigma_{\text{D}}^{- 2}} \right|.$$

Since the achievable rate for the two-hop relaying system is bounded by the minimum of (2) and (4), the achievable rate over the SP $(k,m)$ is given by

5 $${\mathbf{R}}_{k,m} =\frac{\varvec{B}}{2K}\hbox{min} \left({ \log }_{2} \left| {{\mathbf{I}} + {\mathbf{W}}_{{{\text{R}},k}} {\mathbf{H}}_{1,k} {\mathbf{F}}_{{{\text{S}},k}} {\mathbf{F}}_{{{\text{S}},k}}^{H} {\mathbf{H}}_{1,k}^{H} {\mathbf{W}}_{{{\text{R}},k}}^{H} \sigma_{\text{R}}^{- 2}} \right|,{ \log }_{2} \left| {{\mathbf{I}} + {\mathbf{W}}_{{{\text{D}},m}} {\mathbf{H}}_{2,m} {\mathbf{F}}_{{{\text{R}},m}} {\mathbf{F}}_{{{\text{R}},m}}^{H} {\mathbf{H}}_{2,m}^{H} {\mathbf{W}}_{{{\text{D}},m}}^{H} \sigma_{\text{D}}^{- 2}} \right|\right),$$

where $\varvec{B}$ denotes the total bandwidth of the OFDM system and 1/2 results from the transmission duty cycle loss in half duplex two-hop systems.

It has been proved in (Ryu and Choi 2011) that the optimization problem of maximizing the achievable rate for a MIMO DF relaying system can be solved by performing a singular value decomposition (SVD) on ${\mathbf{H}}_{1,k}$ and ${\mathbf{H}}_{2,m}$ to decompose the MIMO channel into multiple parallel independent subchannels with different gain. Due to the full channel state information (CSI) at the nodes, we can use the SVD of the channel matrices to determine transmit-and-receive beamforming matrices at each node. Specifically, the SVD of the channel matrices is given by

6 $${\mathbf{H}}_{i,q} = {\mathbf{U}}_{i,q} {\varvec{\Lambda}}_{i,q} {\mathbf{V}}_{i,q}^{H},$$

where $q = k$ for $i = 1$, and $q = m$ for $i = 2$. Both ${\mathbf{U}}_{i,q}$ and ${\mathbf{V}}_{i,q}^{H}$ are unitary, and ${\varvec{\Lambda}}_{i,q} \in$ ℂ $^{{{\text{Rank}}({\mathbf{H}}_{i,q}) \times {\text{Rank}}({\mathbf{H}}_{i,q})}}$ is a diagonal matrix whose diagonal elements $\{\sqrt {\lambda_{i,l}} \}_{l = 1}^{{{\text{Rank}}({\mathbf{H}}_{i,q})}}$ are nonzero singular values of ${\mathbf{H}}_{i,q}$ in descending order.

By adopting the above SVD of channel matrix ${\mathbf{H}}_{i,q}$, in order to obtain the parallel single-input single-output (SISO) paths, we further choose the precoding matrix at S, the forwarding matrix at R and the receiver filters deployed at R and D as ${\mathbf{F}}_{{{\text{S}},k}} = \sqrt {P_{{{\text{S}},k}}} {\mathbf{V}}_{ 1,k}$, ${\mathbf{F}}_{{{\text{R}},m}} = \sqrt {P_{{{\text{R}},m}}} {\mathbf{V}}_{ 2,m}$, ${\mathbf{W}}_{{{\text{R}},k}} = {\mathbf{U}}_{ 1,k}^{H}$ and ${\mathbf{W}}_{{{\text{D}},m}} = {\mathbf{U}}_{ 2,m}^{H}$, respectively, where $P_{{{\text{S}},k}}$ and $P_{{{\text{R}},m}}$ denote the available transmit power at S over the $k{\text{th}}$ subcarrier and at ${\text{R}}$ over the $m{\text{th}}$ subcarrier, respectively.

Substituting (6) and the above designed matrices into (5), the achievable rate over the SP $(k,m)$ can be rewritten as

7 $${\mathbf{R}}_{k,m} =\frac{\varvec{B}}{2K}\hbox{min} \left({ \log }_{2} \left| {{\mathbf{I}} + P_{{{\text{S}},k}} {\varvec{\Lambda}}_{1,k} {\varvec{\Lambda}}_{1,k}^{H} \sigma_{\text{R}}^{- 2}} \right|,{ \log }_{2} \left| {{\mathbf{I}} + P_{{{\text{R}},m}} {\varvec{\Lambda}}_{2,m} {\varvec{\Lambda}}_{2,m}^{H} \sigma_{\text{D}}^{- 2}} \right|\right).$$

Through the above mentioned operations, the OFDM subcarrier over the two hops are decomposed into multiple available end-to-end (E2E) subchannels, and the number *N* of available spatial subchannels per OFDM subcarrier over the two hops is bounded to the minimum number of spatial subchannels of each hop, specifically, $N = \hbox{min} \{{\text{Rank}}({\mathbf{H}}_{1,k}),{\text{Rank}}({\mathbf{H}}_{2,m})\} = \hbox{min} \{N_{\text{S}},N_{\text{R}},N_{\text{D}} \}$. Since there are *K* subcarriers, the total number of effective E2E subchannels in the MIMO–OFDM system is *KN*. We introduce the subscript $n\,{\mathop{=}\limits^{\triangle}}\,(k-1)K+l$ and $n^{\prime} \, {\mathop{=}\limits^{\triangle}} \, (m-1)K + l^{\prime}$ to simplify the notation, where $1 \le l,l^{\prime} \le N$. As a result, $1 \le n,n^{\prime} \le KN$, and the above mentioned SP $(k,m)$ can be rewritten as SP $(n,n^{\prime})$ which means that the $n$th subchannel over hop 1 is paired with the $n^{\prime}$th subchannel over hop 2. Further, let $\varvec{P}_{\text{S}}$ and $\varvec{P}_{\text{R}}$ be the available transmit power at ${\text{S}}$ and ${\text{R}}$ respectively, we define $P_{{{\text{S}},n}}\mathop{=}\limits^{\triangle} \varvec{P}_{\text{S}} \mu_{n}$, $P_{{{\text{R}},n^{\prime}}}\mathop{=}\limits^{\triangle}\varvec{P}_{\text{R}} \overline{\mu}_{n\prime}$, where $\mu_{n} \in [0,1]$ and $\overline{\mu}_{n\prime} \in [0,1]$ denote the power allocating factor at S for subchannel $n$ over hop-1 and the power allocating factor at R for subchannel $n^{\prime}$ over hop-2, respectively. Consequently, the achievable rate over SP $(n,n^{\prime})$ can be expressed as

8 $$R_{{n,n^{\prime}}} =\frac{\varvec{B}}{2K}\hbox{min} \left({{ \log }_{2} (1 + \frac{{P_{{{\text{S}},n}} \lambda_{1,n}}}{{\sigma_{\text{R}}^{2}}}),{ \log }_{2} (1 + \frac{{P_{{{\text{R}},n^{\prime}}} \lambda_{{2,n^{\prime}}}}}{{\sigma_{\text{D}}^{2}}})} \right).$$

### The TSDFR protocol and optimization problem formulation

In this section, we shall first propose the transmission protocol which enable the simultaneous information and power transfer for the MIMO–OFDM DF relaying system by adopting the TS receiver architecture proposed in (Zhou et al. 2013), and then we formulate the optimization problem to maximize the system achievable rate.^1^

#### Protocol description for TSDFR

**Fig. 1:**
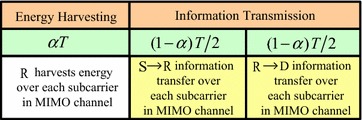
Illustration of the proposed TSDFR protocol

Figure 1 depicts the main transmission process in the proposed TSDFR protocol. By considering the time switching receiver architecture described in (Zhou et al. 2013), the proposed TSDFR protocol consists of three phases: the energy transfer phase, the information transmission from S phase and the information relaying from R phase, as shown in Fig. 1. For a time period *T*, the time durations assigned to each phase are $\alpha T$, $(1 - \alpha)T/2$ and $(1 - \alpha)T/2$, respectively, where $0 \le \alpha \le 1$ denotes the time assignment factor.

In the first phase,^2^ S transfers energy to R, and the received signal at R for energy harvesting is given as follows

9 $${\mathbf{y}}_{{{\text{R}},k}}^{{ ( {\text{EH)}}}} ={\mathbf{H}}_{1,k} \varvec{x}_{k} + {\mathbf{n}}_{{{\text{R}},k}},$$

where $\varvec{x}_{k}$ denotes the transmitted signal vector for energy transfer over subcarrier *k*. Thus, the harvested energy at R over subcarrier *k* can be given as

10 $$E_{{{\text{R}},k}} =\alpha \eta \left\| {{\mathbf{H}}_{1,k} \varvec{x}_{k}} \right\|^{2},$$

where $0 < \eta \le 1$ denotes the energy conversion efficiency. Thus, the total harvested energy over $K$ subcarriers can be given by $E^{{({\text{TSDFR}})}} = \sum\nolimits_{k = 1}^{K} {E_{{{\text{R}},k}}}$, and note that, the total transferred energy is limited by the available power at S, i.e. $\sum\nolimits_{k = 1}^{K} \text{tr}(\varvec{x}_{k} \varvec{x}_{k}^{H}) \le \varvec{P}_{\varvec{s}}$. We assume that all the harvested energy in the first phase is used to relay the information in the third phase, so the available transmit power at R in the information relaying phase is given by

11 $$\varvec{P}_{\varvec{R}} = \frac{{E^{{({\text{TSDFR}})}}}}{(1 - \alpha)T/2} = \frac{2\alpha \eta}{1 - \alpha}\sum\limits_{k = 1}^{K} {\left\| {{\mathbf{H}}_{1,k} \varvec{x}_{k}} \right\|^{2}}.$$

In the second phase, i.e. the information transmission from S phase, S delivers the signal vector $\varvec{s}_{k} \in{\mathbb{C}}^{{N_{\text{S}} \times 1}}$ to R, the received signal and the achievable rate at R are the same with (1) and (2). In the third phase, i.e. the information relaying from R phase, R decodes the signal $\varvec{s}_{k}$ received from S and forwards it to D over all *KN* subchannels.

Further, we define $P_{{{\text{S}},n}}\mathop{=}\limits^{\triangle}\varvec{P}_{\text{S}} \mu_{n}$, $P_{{{\text{R}},n^{\prime}}}\mathop{=}\limits^{\triangle}\varvec{P}_{\text{R}} \overline{\mu}_{n\prime}$, and the achievable rate $R_{n,n\prime}$ over SP $(n,n\prime)$ in (8) can be expressed as

12 $$R_{{n,n^{\prime}}}^{{({\text{TSDFR}})}} =\frac{{(1 - \alpha)\varvec{B}}}{2K}\hbox{min} \left({{ \log }_{2} \left(1 + \frac{{\varvec{P}_{\text{S}} \mu_{n} \lambda_{1,n}}}{{\sigma_{\text{R}}^{2}}}\right),{ \log }_{2} \left(1 + \frac{{\varvec{P}_{\text{R}} \overline{\mu}_{{n^{\prime}}} \lambda_{{2,n^{\prime}}}}}{{\sigma_{\text{D}}^{2}}}\right)} \right).$$

#### Optimization problem formulation for TSDFR

The achievable rate of the TSDFR protocol in the MIMO–OFDM DF relaying system can be given by

13 $$C^{{({\text{TSDFR}})}} = \sum\limits_{n = 1}^{KN} {\sum\limits_{{n^{\prime} = 1}}^{KN} {\theta_{{n,n^{\prime}}} R_{{n,n^{\prime}}}^{{({\text{TSDFR}})}}}},$$

where $\theta_{{n,n^{\prime}}} \in \{0,1\}$ denotes the subchannel-paring. Specifically, $\theta_{{n,n^{\prime}}} = 1$ means that the $n$th subchannel over hop-1 is paired with the $n^{\prime}$th subchannel over hop-2. Otherwise, $\theta_{{n,n^{\prime}}} = 0$. Let ${\mathbf{X}}_{k} =E\{\varvec{x}_{k} \varvec{x}_{k}^{H} \}$ denote the covariance matrix of $\varvec{x}_{k}$, ${\mathbf{X}}_{\text{S}} = \{{\mathbf{X}}_{ 1},{\mathbf{X}}_{ 2},\ldots,{\mathbf{X}}_{k} \}$ indicates the energy transfer pattern at S. Thus, the optimization problem of maximizing the achievable rate for a MIMO–OFDM DF relaying system can be formulated as follows

14a $$\mathop {\max}\limits_{{{\mathbf{X}}_{\text{S}},\varvec{\mu},\overline{\varvec{\mu}},\varvec{\theta},\alpha}} C^{{({\text{TSDFR}})}}$$

14b $$s.t.\sum\limits_{k = 1}^{K} {\text{tr(}\varvec{x}_{k}} \varvec{x}_{k}^{H}) \le \varvec{P}_{\varvec{s}},{\mathbf{X}}_{i} \succ 0,$$

14c $$\sum\limits_{n = 1}^{KN} {\mu_{n} \le 1},\sum\limits_{{n^{\prime} = 1}}^{KN} {\bar{\mu}_{{n^{\prime}}} \le 1},\mu_{n} \in [0,1],\bar{\mu}_{{n^{\prime}}} \in [0,1], \quad \forall n, \quad \forall n^{\prime},$$

14d $$\sum\limits_{n = 1}^{KN} {\theta_{{n,n^{\prime}}} = 1},\sum\limits_{{n^{\prime} = 1}}^{KN} {\theta_{{n,n^{\prime}}} = 1},\theta_{{n,n^{\prime}}} \in \{0,1\}, \quad \forall n, \quad \forall n^{\prime},$$

14e $$0 \le \alpha \le 1$$

where (14b) indicates that the energy transferred in the first phase is restricted by the available power $\varvec{P}_{\text{S}}$ at S, (14c) actually means that the available transmit power at S and R are constrained by $\varvec{P}_{\text{S}}$ and $\varvec{P}_{\text{R}}$. (14d) indicates that each subchannel of hop-1 can only be paired with one subchannel of hop-2.

### The PSDFR protocol and optimization problem formulation

#### Protocol description for PSDFR

**Fig. 2:**
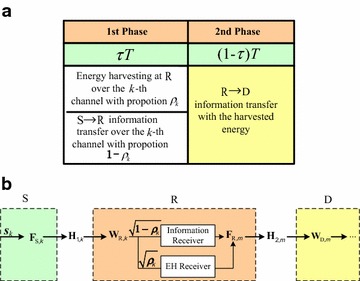
**a** Illustration of the proposed PSDFR protocol. **b** The structure of PSDFR

The framework of the PSDFR protocol is illustrated in Fig. 2a. The total time period *T* is divided into two phases, where the first phase lasts for a time duration of $\tau T$, and the second one lasts for a time duration of $(1 - \tau)T$, where $0 < \tau < 1$ denotes the time division pattern between the two phases in the protocol. In practice, $\tau$ can be designed as different values to satisfy the transport protocol. In the first phase, S transmits a signal vector to R over all the *KN* subchannels, where both energy and information are carried on this signal vector, so that R can harvest energy from the signal. In the second phase, R decodes the information received from S, and uses all the harvested energy to forward the information to D.

For clarity, we depict the structure of our proposed PSDFR protocol in Fig. 2b. Let $\varvec{\rho}$ be the power splitting factor matrix, which denotes the portion of power split to the EH receiver. The received signals are first processed at R by its receiver filter ${\mathbf{W}}_{{{\text{R}},k}}$, and then split into two flows. Specifically, the $(1 -\varvec{\rho})$ part is input into the information receiver for the information decoding, whereas the remaining part $\varvec{\rho}$ is input into the EH receiver for energy harvesting, and all the harvested energy is then allocated to R’s forwarding matrix ${\mathbf{F}}_{{{\text{R}},m}}$. The detailed transmission process of PSDFR is as follows.

In the first phase, S transmits a signal vector to R, and the harvested energy at R over subcarrier $k$ can be given as

15 $$E_{{{\text{R}},k}} =\eta \tau T\left\| {\varvec{\rho}_{k}^{1/2} {\mathbf{W}}_{{{\text{R}},k}} {\mathbf{H}}_{1,k} {\mathbf{F}}_{{{\text{S}},k}} \varvec{s}_{k}} \right\|^{2},$$

where $\varvec{\rho}_{k} = {\text{diag}}\{\rho_{k,1},\rho_{k,2},\ldots,\rho_{k,N} \}$, $0 \le \rho_{k,i} \le 1$. By adopting the SVD operation of ${\mathbf{H}}_{1,k}$ similar to (6), and choose ${\mathbf{F}}_{{{\text{S}},k}} = \sqrt {P_{{{\text{S}},k}}} {\mathbf{V}}_{ 1,k}$ and ${\mathbf{W}}_{{{\text{R}},k}} = {\mathbf{U}}_{ 1,k}^{H}$, (15) can be rewritten as

16 $$E_{{{\text{R}},k}} =\eta \tau TP_{{{\text{S}},k}} \left\| {\varvec{\rho}_{k}^{1/2} {\varvec{\Lambda}}_{1,k}} \right\|^{2} = \tau T\sum\limits_{i = 1}^{N} {\eta \rho_{k,i} P_{{{\text{S}},k}} \lambda_{1,k}^{(i)}},$$

where $P_{{{\text{S}},k}}$ denotes the available transmit power at S over subcarrier $k$, and $\eta \rho_{k,i} P_{{{\text{S}},k}} \lambda_{1,k}^{(i)}$ can be regarded as the energy harvested on the $i$th subchannel over the $k$th subcarrier. By introducing the subscript $n \, {\mathop{=}\limits^{\triangle}}\, (k - 1)K + l$, the total energy harvested at R in phase 1 can be given as follows

17 $$E^{{ ( {\text{PSDFR)}}}} =\tau T\sum\limits_{k = 1}^{K} {\sum\limits_{i = 1}^{N} {\eta \rho_{k,n} P_{{{\text{S}},k}} \lambda_{1,k}^{(i)}}} = \tau T\sum\limits_{n = 1}^{KN} {\eta \rho_{n} \lambda^{1,n} \varvec{P}_{\text{S}}} \mu_{n},$$

where $\mu_{n} \in [0,1]$ denotes the power allocating factor at S for subchannel $n$.

Meanwhile, after the processing of the information receiver, the sampled baseband signal at ${\text{R}}$ in phase 1 can be given by

18 $${\mathbf{y}}_{{{\text{R}},k}} =(\varvec{I} -\varvec{\rho})^{1/2} {\mathbf{W}}_{{{\text{R}},k}} {\mathbf{H}}_{1,k} {\mathbf{F}}_{{{\text{S}},k}} \varvec{s}_{k} + {\mathbf{n}}_{{{\text{R}},k}}.$$

In the second phase, R decodes the signal $\varvec{s}_{k}$ received from S and forwards it to D over all $KN$ subchannels. The received signal and the achievable rate at D are the same with (3) and (4).

In this paper, we adopt such a power splitting strategy that, the energy harvested over the $n$th subchannel of hop-1 is all used for the information relaying over its paired subchannel $n^{\prime}$ of hop-2. So the available transmit power at R for $n^{\prime}$th subchannel is $P_{{{\text{R}},n^{\prime}}} \mathop{=}\limits^{\triangle} \frac{{\tau T\eta \rho_{n} \lambda_{1,n} \varvec{P}_{\text{S}} \mu_{n}}}{{(1{-}\tau)T}} =\frac{\tau}{{1{-}\tau}}\eta \rho_{n} \lambda_{1,n} \varvec{P}_{\text{S}} \mu_{n}$. Since $P_{{{\text{S}},n}} \mathop{=}\limits^{\triangle} \eta (1{-}\rho_{n})\varvec{P}_{\text{S}} \mu_{n}$, the achievable rate $R_{{n,n^{\prime}}}$ over SP $(n,n^{\prime})$ in (8) can be rewritten as

19 $$R_{{n,n^{\prime}}}^{{({\text{PSDFR}})}} =\frac{{\tau \varvec{B}}}{K}\hbox{min} \left({{ \log }_{2} (1 + \frac{{(1 - \rho_{n})\varvec{P}_{\text{S}} \mu_{n} \lambda_{1,n}}}{{\sigma_{\text{R}}^{2}}}),{ \log }_{2} (1 + \frac{\tau}{{1{-}\tau}} \cdot \frac{{\eta \rho_{n} \varvec{P}_{\text{S}} \mu_{n} \lambda_{1,n} \lambda_{{2,n^{\prime}}}}}{{\sigma_{\text{D}}^{2}}})} \right) .$$

#### Optimization problem formulation for PSDFR

The achievable rate of the PSDFR protocol in the DF relaying system can be given by

20 $$C^{{({\text{PSDFR}})}} = \sum\limits_{n = 1}^{KN} {\sum\limits_{{n^{\prime} = 1}}^{KN} {\theta_{{n,n^{\prime}}} R_{{n,n^{\prime}}}^{{({\text{PSDFR}})}}}},$$

where $\theta_{{n,n^{\prime}}} \in \{0,1\}$ denotes the subchannel-paring pattern, and was illustrated below (13). Thus, the optimization problem of maximizing the achievable rate for a MIMO–OFDM DF relaying system employing PSDFR can be formulated by

21a $$\mathop {\max}\limits_{{\varvec{\rho},\varvec{\theta},\varvec{\mu}}} C^{{({\text{PSDFR}})}}$$

21b $$s.t.\sum\limits_{n = 1}^{KN} {\mu_{n} \le 1},\mu_{n} \in [0,1], \quad\forall n,$$

21c $$\sum\limits_{n = 1}^{KN} {\theta_{{n,n^{\prime}}} = 1},\sum\limits_{{n^{\prime} = 1}}^{KN} {\theta_{{n,n^{\prime}}} = 1},\theta_{{n,n^{\prime}}} \in \{0,1\},\quad \forall n,\forall n^{\prime},$$

21d $$0 \le \rho_{n} \le 1,\quad \forall n \in \{1,2, \ldots,KN\}$$

where (21b) indicates that the available transmit power at S is constrained by $\varvec{P}_{\text{S}}$, and (21c) indicates that each subchannel of hop-1 can only be paired with one subchannel of hop-2.

### Achievable rate optimization

#### Achievable rate optimization for TSDFR

It can be observed that the problem in (14) is a combinatorial optimization problem with high computational complexity due to the discrete parameter $\theta_{{n,n^{\prime}}} \in \{0,1\}$, so it is difficult to be solved by conventional methods. The main ideas to solve (14) are exhibited in Algorithm 1: Firstly, only energy is delivered in the first phase, which means that only ${\mathbf{X}}_{\text{S}}$ needs to be optimized and it is independent with other variables. Thus, we could design ${\mathbf{X}}_{\text{S}}$ independently. Secondly, according to the separation principle designed in (Hajiaghayi et al. 2011), the joint channel pairing and power allocation optimization problem can be decoupled into two separate sub-problems. So, we can optimize $\theta$ independently without considering other variables. Thirdly, based on the optimal ${\mathbf{X}}_{\text{S}}^{\#}$ and $\varvec{\theta}^{\#}$, we propose an ALPF algorithm to jointly optimize $\varvec{\mu}_{n}$, $\overline{\varvec{\mu}}_{{n^{\prime}}}$ and $\alpha$ to maximize $C^{{({\text{TSDFR}})}}$. The details of Algorithm 1 are described in the successive subsections.
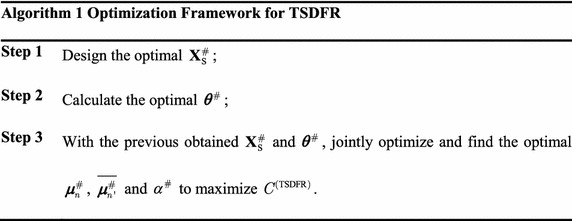

1. *Optimal*${\mathbf{X}}_{\text{S}}^{\#}$*for TSDFR:*

It can be seen from Eq. (11) that for a given $\alpha$, the larger $E^{{({\text{TSDFR}})}}$, the higher $\varvec{P}_{\text{R}}$, that is to say, there will be much more available transmit power for information delivery in the second phase. As a result, for a given $\alpha$, the optimization problem can be translated into (22):

22 $$\begin{aligned} & \mathop {\max}\limits_{{{\mathbf{X}}_{\text{S}}}} \left\| {{\mathbf{H}}_{1,k} \varvec{x}_{k}} \right\|^{2} \hfill \\ & s.t.\sum\limits_{k = 1}^{K} {{\text{tr}}({\mathbf{X}}_{k}) \le \varvec{P}_{\varvec{s}},} {\mathbf{X}}_{i} \succ 0 \hfill \\ \end{aligned}$$

For the above Problem (22), (Xiong et al. 2015) has given the optimal solutions and the corresponding proof. Firstly, by performing the SVD on ${\mathbf{H}}_{1,k}$ and ${\mathbf{X}}_{k}$, we obtain ${\mathbf{H}}_{1,k} = {\mathbf{U}}_{1,k} {\varvec{\Lambda}}_{1,k} {\mathbf{V}}_{1,k}^{H}$ and ${\mathbf{X}}_{k} = {\mathbf{V}}_{\varvec{X}}^{(k)} {\varvec{\Lambda}}_{\varvec{X}}^{(k)} {\mathbf{V}}_{\varvec{X}}^{(k)H}$, where $\nu_{1,1}^{(k)}$ denotes the first column of ${\mathbf{V}}_{1,k}$, and ${\mathbf{V}}_{\varvec{X}}^{(k)} = [\varvec{\nu}_{{\varvec{X},1}}^{(k)},\varvec{\nu}_{{\varvec{X},2}}^{(k)},\ldots,\varvec{\nu}_{{\varvec{X},{\text{Rank}}({\mathbf{H}}_{1,k})}}^{(k)}]$, $\overline{{\mathbf{H}}}_{1,k} {\mathbf{= H}}_{1,k} {\mathbf{V}}_{\varvec{X}}^{(k)} = [\widehat{h}_{1,1}^{(k)},\widehat{h}_{1,2}^{(k)},\ldots,\widehat{h}_{{1,{\text{Rank}}({\mathbf{H}}_{1,k})}}^{(k)}]$. According to the Theorem 1 in (Xiong et al. 2015), the optimal solution^3^ for ${\mathbf{X}}_{\text{S}}^{\#}$ is ${\mathbf{X}}_{\text{S}}^{\#} = \{{\mathbf{X}}_{k}^{\#} \}$, where

23 $${\mathbf{X}}_{k}^{\#} = \left\{{\begin{array}{ll} {\varvec{P}_{\varvec{s}} \nu_{1,1}^{(k)} \nu_{1,1}^{(k)H},} & {k = \arg \mathop {\max}\limits_{a = 1,2,\ldots,K} \left\| {\widehat{h}_{1,1}^{(a)}} \right\|^{2},} \hfill \\ {0,} & {\text{otherwise}} \hfill \\ \end{array}} \right.$$

For a given $\alpha$, the optimal $\varvec{P}_{\text{R}}$ can be obtained as $\varvec{P}_{\text{R}}^{*} = \frac{2\alpha \eta}{1 - \alpha}\varvec{P}_{\text{S}} \left\| {\widehat{h}_{1,1}^{(u)}} \right\|^{2}$, where $u = \arg \mathop {\max}\limits_{a = 1,2,\ldots,K} \left\| {\widehat{h}_{1,1}^{(a)}} \right\|^{2}$.

(2) *Optimal*$\varvec{\theta}^{\#}$*for TSDFR:*

For a given $\alpha$, substituting the obtained ${\mathbf{X}}_{\text{S}}^{\#}$ into Problem (14), the optimization problem can be rewritten in (24). And as described in (8), the signal transmitted over the $n$th subchannel in the first hop is supposed to be delivered over the $n^{\prime}$th subcarrier in the second hop, which is also known as the subchannel pairing between the two hops. Further, we described the optimal subchannel pairing in Theorem 1 as follows.

24 $$\begin{aligned} & \mathop {\max}\limits_{{\mu,\overline{\mu},\theta}} C^{(TSDFR)} \\ & s.t.\sum\limits_{n = 1}^{KN} {\mu_{n} \le 1},\sum\limits_{{n^{\prime} = 1}}^{KN} {\overline{\mu}_{{n^{\prime}}} \le 1},\mu_{n} \in [0,1],\quad\overline{\mu}_{{n^{\prime}}} \in [0,1],\quad \forall n, \forall n^{\prime}, \\ & \quad \sum\limits_{n = 1}^{KN} {\theta_{{n,n^{\prime}}} = 1},\sum\limits_{{n^{\prime} = 1}}^{KN} {\theta_{{n,n^{\prime}}} = 1}, \quad \theta_{{n,n^{\prime}}} \in \{0,1\}, \quad \forall n, \forall n^{\prime} \\ \end{aligned}$$

##### **Theorem 1**

*The optimal subchannel pairing*$\varvec{\theta}^{\#}$*is performed in the order of sorted channel gain which means that the subchannel with*$i$th ($i =1,2, \ldots,KN$) *largest channel gain (normalized against the noise power) over hop-1 should be paired with the subchannel with*$i$th *largest channel gain (also normalized against the noise power) over hop-2*.

##### *Proof*

According to the separation principle in (Hajiaghayi et al. 2011), the joint channel pairing and power allocation optimization problem can be operated in a separated manner, and the optimal channel pairing is performed individually at the relay in the order of sorted channel gain. Note that, different ${\mathbf{X}}_{\text{S}}$ or $\alpha$ only affect the relay’s available power in the third phase, rather than the channel gain of all subchannels. Thus, $\varvec{\theta}^{\#}$ is global optimal for Problem (14).$\square$

(3) *Optimal*$\varvec{\mu}_{n}^{\#}$, $\overline{{\varvec{\mu}_{{n^{\prime}}}^{\#}}}$ and $\alpha^{\#}$*for TSDFR:*

According to the max-flow min-cut theorem (Liang 2006), the achievable rate of each subchannel is limited by the minimal rate over the two hops. Thus, the rates of two hops are equal to each other when $C^{{({\text{TSDFR}})}}$ is maximized. Further, by substituting the optimal ${\mathbf{X}}_{\text{S}}^{\#}$ and $\varvec{\theta}^{\#}$ into Problem (14), the original optimization problem can be rewritten as follows

25 $$\begin{aligned} & \mathop {\max}\limits_{{\varvec{\mu},\overline{\varvec{\mu}},\alpha}}\, \frac{{(1 - \alpha)\varvec{B}}}{2K}\sum\limits_{n = 1}^{KN} {{ \log }_{2} \left({1 + \frac{{\varvec{P}_{\text{S}} \mu_{n} \lambda_{1,n}}}{{\sigma_{\text{R}}^{2}}}} \right)} \\ & s.t.\, \sum\limits_{n = 1}^{KN} {\mu_{n} \le 1},\sum\limits_{{n^{\prime} = 1}}^{KN} {\overline{\mu}_{{n^{\prime}}} \le 1},\mu_{n} \ge 0,\quad\overline{\mu}_{{n^{\prime}}} \ge 0 \\ & \quad \frac{{\varvec{P}_{\text{S}} \mu_{n} \lambda_{1,n}}}{{\sigma_{\text{R}}^{2}}} =\frac{{\varvec{P}_{\text{R}} \overline{\mu}_{{n^{\prime}}} \lambda_{{2,n^{\prime}}}}}{{\sigma_{\text{D}}^{2}}},\quad for\;\theta_{{n,n^{\prime}}} = 1, \\ & \quad 0 \le \alpha \le 1 \\ \end{aligned}$$

It can be observed that the problem in (25) is still a nonlinear and non-convex optimization problem which is difficult to solve. Here, we introduce an ALPF algorithm to find the jointly optimal $\varvec{\mu}_{n}^{\#}$, $\overline{{\varvec{\mu}_{{n^{\prime}}}^{\#}}}$ and $\alpha^{\#}$. The ALPF method is a classical method to solve nonlinear optimization problem with constraints, which can ensure the local optimality of solutions, and even the global optimality for convex problems. Moreover, various methods can be used to solve the sub-problems in ALPF, which makes it relatively independent and adaptive (Bhatti 2000).

The basic idea of ALPF is to convert the constrained original problem into an unconstrained problem by adding a penalty term to the Lagrangian function of the original problem. And the inequality constraints of the original problem can also be converted to equality constraints through the addition of slack variables (Ramamonjison and Bhargava 2012; Fodor et al. 2009; Reider and Fodor 2010; Reider et al. 2010). First we write the problem in (25) into standard formulation as follows

26 $$\begin{aligned}& \mathop {\min}\limits_{{\varvec{\mu},\overline{\varvec{\mu}},\alpha}} \;\frac{{(\alpha - 1)\varvec{B}}}{2K}\sum\limits_{n = 1}^{KN} {{ \log }_{2} \left({1 + \frac{{\varvec{P}_{\text{S}} \mu_{n} \lambda_{1,n}}}{{\sigma_{\text{R}}^{2}}}} \right)} \\ & s.t.\;\sum\limits_{n = 1}^{KN} {\mu_{n} +S_{1} = 1},\sum\limits_{{n^{\prime} = 1}}^{KN} {\overline{\mu}_{{n^{\prime}}} +S_{2} = 1},\mu_{n} \ge 0,\quad\overline{\mu}_{{n^{\prime}}} \ge 0,S_{1} \ge 0,S_{2} \ge 0, \\ & \quad \frac{{\varvec{P}_{\text{S}} \mu_{n} \lambda_{1,n}}}{{\sigma_{\text{R}}^{2}}} =\frac{{\varvec{P}_{\text{R}} \overline{\mu}_{{n^{\prime}}} \lambda_{{2,n^{\prime}}}}}{{\sigma_{\text{D}}^{2}}},\quad for\;\theta_{{n,n^{\prime}}} = 1, \hfill \\ & \quad 0 \le \alpha \le 1 \hfill \\ \end{aligned}$$

The unconstrained augmented Lagrangian penalty function of (26) can be written as

27 $$\begin{aligned}& P\left({\varvec{x},\varvec{\xi},\varvec{\delta}} \right) = \frac{{(\alpha - 1)\varvec{B}}}{2K}\sum\limits_{n = 1}^{KN} {{ \log }_{2} \left({1 + A_{n} \mu_{n}} \right)} - \xi_{1} \left(\sum\limits_{n = 1}^{KN} {\mu_{n} +S_{1} - 1}\right) - \xi_{2} \left(\sum\limits_{{n^{\prime} = 1}}^{KN} {\overline{\mu}_{{n^{\prime}}} +S_{2} - 1}\right) \\&\quad - \sum\limits_{n = 1}^{KN} {\xi_{{n,n^{\prime}}} \theta_{{n,n^{\prime}}} \left({A_{n} \mu_{n} - \frac{2\alpha}{1 - \alpha}B_{{n^{\prime}}} \overline{\mu}_{{n^{\prime}}}} \right)+\frac{1}{2}\delta_{1} \left(\sum\limits_{n = 1}^{KN} {\mu_{n} +S_{1} - 1}\right)^{2} + \frac{1}{2}\delta_{2} \left(\sum\limits_{{n^{\prime} = 1}}^{KN} {\overline{\mu}_{{n^{\prime}}} +S_{2} - 1}\right)^{2}} \\ &\quad+ \sum\limits_{n = 1}^{KN} {\frac{1}{2}} \delta_{{n,n^{\prime}}} \theta_{{n,n^{\prime}}} \left({A_{n} \mu_{n} - \frac{2\alpha}{1 - \alpha}B_{{n^{\prime}}} \overline{\mu}_{{n^{\prime}}}} \right)^{2}, \hfill \\ \end{aligned}$$

where $A_{n} = \frac{{\varvec{P}_{\text{S}} \lambda_{1,n}}}{{\sigma_{\text{R}}^{2}}}$, $B_{{n^{\prime}}} = \frac{{\varvec{P}_{\text{R}} \lambda_{{2,n^{\prime}}}}}{{\sigma_{\text{D}}^{2}}}$, $\varvec{x} = \left({\alpha,\varvec{\mu},\overline{\varvec{\mu}},S_{1},S_{2}} \right)$, $S_{1}$ and $S_{2}$ are positive slack variables, $\varvec{\xi}$ and $\varvec{\delta}$ denote the Lagrangian multipliers and the penalty parameter, respectively, and $\varvec{\xi}= (\xi_{1},\xi_{2},\xi_{{n,n^{\prime}}})$, $\varvec{\delta}= (\delta_{1},\delta_{2},\delta_{{n,n^{\prime}}})$.

In the $k$th iteration, $\varvec{x}^{(k + 1)}$ can be updated as follows

28 $$\varvec{x}^{(k + 1)} = {\text{argmin}}P(\varvec{x}^{(k)},\varvec{\xi}^{(k)},\varvec{\delta}^{(k)}).$$

Then the Lagrange multipliers can be obtained by

$$\xi_{1}^{(k+1)} =\xi_{1}^{(k)} {-}\delta_{1}^{(k)} \left({\sum\limits_{n = 1}^{KN} {\mu_{n}^{(k+1)} +S_{1}^{(k+1)} - 1}} \right),$$

29 $$\begin{aligned} \xi_{2}^{(k+1)} &=\xi_{2}^{(k)} - \delta_{2}^{(k)} \left({\sum\limits_{{n^{\prime} = 1}}^{KN} {\overline{\mu}_{{n^{\prime}}}^{(k+1)} +S_{2}^{(k+1)} - 1}} \right), \hfill \\ \xi_{{n,n^{\prime}}}^{(k+1)} &=\xi_{{n,n^{\prime}}}^{(k)} - \delta_{{n,n^{\prime}}}^{(k)} \left({A_{n} \mu_{n}^{(k+1)} - \frac{{2\alpha^{(k+1)}}}{{1 - \alpha^{(k+1)}}}B_{{n^{\prime}}}^{(k+1)} \overline{\mu}_{{n^{\prime}}}^{(k+1)}} \right). \hfill \\ \end{aligned}$$

And the penalty parameters can be updated by

30 $$\delta_{1}^{(k + 1)} = \left \{\begin{array}{ll} \delta_{1}^{(k)}, & \quad {\text{if}}\; \left| \sum\limits_{n = 1}^{KN} \mu_{n}^{(k+1)} +S_{1}^{(k+1)} - 1 \right| \le \frac{1}{4}\left| \sum\limits_{n = 1}^{KN} \mu_{n}^{(k)} +S_{1}^{(k)} - 1 \right| \\ \hbox{max} \{10 \delta_{1}^{(k)},k^{2} \}, & \quad {\text{otherwise}}. \\ \end{array} \right. ,$$

31 $$\delta_{2}^{(k + 1)} = \left\{{\begin{array}{ll} {\delta_{2}^{(k)},} &\quad {{\text{if}}\; \left| {\sum\limits_{{n^{\prime} = 1}}^{KN} {\overline{\mu}_{{n^{\prime}}}^{(k+1)} +S_{2}^{(k+1)} - 1}} \right| \le \frac{1}{4}\left| {\sum\limits_{{n^{\prime} = 1}}^{KN} {\overline{\mu}_{{n^{\prime}}}^{(k)} +S_{2}^{(k)} - 1}} \right|} \\ {\hbox{max} \{10\delta_{2}^{(k)},k^{2} \},} & \quad {{\text{otherwise}}.} \\ \end{array}} \right.,$$

32 $$\delta_{{n,n^{\prime}}}^{(k + 1)} = \left\{{\begin{array}{ll} {\delta_{{n,n^{\prime}}}^{(k)},} & \quad {{\text{if}} \; \left| {A_{n} \mu_{n}^{(k + 1)} - \frac{{2\alpha^{(k + 1)}}}{{1 - \alpha^{(k + 1)}}}B_{{n^{\prime}}}^{(k + 1)} \overline{\mu}_{{n^{\prime}}}^{(k + 1)}} \right| \le \frac{1}{4}\left| {A_{n} \mu_{n}^{(k)} - \frac{{2\alpha^{(k)}}}{{1 - \alpha^{(k)}}}B_{{n^{\prime}}}^{(k)} \overline{\mu}_{{n^{\prime}}}^{(k)}} \right|} \\ {\hbox{max} \{10\delta_{{n,n^{\prime}}}^{(k)},k^{2} \},} & \quad {\text{otherwise}} \\ \end{array}} \right.$$

By defining the constraint violation function in (33), we present the main steps of ALPF algorithm for TSDFR as shown in Algorithm 2.

33 $$C^{(-)} (\varvec{x}) = \left({\sum\limits_{n = 1}^{KN} {\mu_{n} +S_{1} - 1},\sum\limits_{{n^{\prime} = 1}}^{KN} {\overline{\mu}_{{n^{\prime}}} +S_{2} - 1},A_{n} \mu_{n} - \frac{2\alpha}{1 - \alpha}B_{{n^{\prime}}} \overline{\mu}_{{n^{\prime}}}} \right).$$

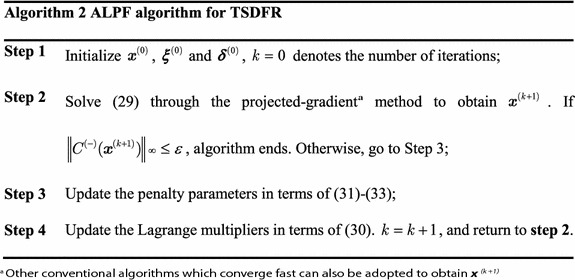


#### Achievable rate optimization for PSDFR

It can be observed that (21) is also a combinatorial optimization problem with the discrete parameters $\theta_{{n,n^{\prime}}} \in \{0,1\}$. The main ideas to solve (21) are as follows: firstly, for a given *τ*, the optimal time assignment factor $\varvec{\rho}^{\#} (\tau)$ could be calculated according to the DF cooperative channel characteristics. Then, by substituting $\varvec{\rho}^{\#} (\tau)$ into (21), the original optimization problem can be simplified to an optimization problem with regard to parameters *τ*, $\varvec{\theta}$ and $\varvec{\mu}$. Secondly, the optimal subchannel pairing pattern $\varvec{\theta}^{\#}$ can be obtained according to the separation principle (Hajiaghayi et al. 2011). Thirdly, the optimal time division pattern $\tau$ and the power allocation scheme $\varvec{\mu}$ at S could be optimized through the ALPF algorithm. The main ideas to solve (21) are exhibited in Algorithm 3, and the details of each step are described in the following subsections.
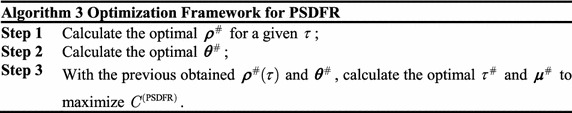

1. *Optimal*$\varvec{\rho}^{\#}$*for PSDFR:*

##### **Theorem 2**

*For a given*$\tau$, *the optimal power splitting factor*$\rho_{n}$*for each subchannel pair*$(n,n^{\prime})$*can be given as follows*

34 $$\rho_{n}^{\#} = \frac{A_{n}}{A_{n} + \frac{\tau}{1 - \tau}D_{n}},\quad n = 1,2,\ldots,KN,$$

where $$A_{n} = \frac{{\varvec{P}_{\text{S}} \lambda_{1,n}}}{{\sigma_{\text{R}}^{2}}}$$, $$D_{n} = \frac{{\eta \varvec{P}_{\text{S}} \lambda_{1,n} \lambda_{{2,n^{^{\prime}}}}}}{{\sigma_{\text{D}}^{2}}}$$.

##### *Proof*

According to the max-flow min-cut theorem (Liang 2006), the achievable rate of each subchannel is limited by the minimal rate over the two hops. Thus, the rates of two hops are equal to each other when $C^{{({\text{PSDFR}})}}$ is maximized, which means that

35 $$R^{{{\text{R}},n}} = {\text{ log}}_{2} \left({1 + \frac{{(1 - \rho_{n})\varvec{P}_{\text{S}} \mu_{n} \lambda_{1,n}}}{{\sigma_{\text{R}}^{2}}}} \right) = R^{{{\text{D}},n^{\prime}}} = { \log }_{2} \left({1 + \frac{\tau}{{1{-}\tau}} \cdot \frac{{\eta \rho_{n} \varvec{P}_{\text{S}} \mu_{n} \lambda_{1,n} \lambda_{{2,n^{\prime}}}}}{{\sigma_{\text{D}}^{2}}}} \right),$$

thus, $(1 - \rho_{n})A_{n} \mu_{n} = \frac{\tau}{{1{-}\tau}} \cdot \rho_{n} D_{n} \mu_{n}$, and the optimal $\rho_{n}$ can be obtained.$\square$

2) *Optimal*$\varvec{\theta}^{\#}$*for PSDFR:*

Because the PS factor $\varvec{\rho}$ and the power allocation scheme $\varvec{\mu}$ do not affect the channel gain of all the subchannels, according to the separation principle in (Hajiaghayi et al. 2011), Theorem 1 still holds for PSDFR, which means that $\varvec{\theta}^{\#}$ is performed in the order of the sorted channel gain.

3) *Optimal*$\tau^{\#}$ and $\varvec{\mu}^{\#}$*for PSDFR:*

By substituting the optimal $\varvec{\rho}^{\#} (\tau)$ and $\varvec{\theta}^{\#}$ obtained from Theorems 2 and 1 into Problem (21), the original optimization problem can be rewritten as follows

36 $$\begin{aligned} & \mathop {\max}\limits_{{\tau,\varvec{\mu}}} \;C^{{({\text{PSDFR}})}} \\ & s.t.\;\sum\limits_{n = 1}^{KN} {\mu_{n} \le 1}, \quad \mu_{n} \in [0,1], \quad \forall n \in \{1,2, \ldots,KN\}, \\ & \quad 0 < \tau < 1 \hfill \\ \end{aligned}$$

It can be observed that the problem in (36) is still a nonlinear and non-convex optimization problem which is difficult to solve. By using the similar ALPF algorithm proposed in the previous subsesction, the problem in (36) can be reformulated into the standard formulation as follows

37 $$\begin{aligned} & \mathop {\min}\limits_{{\tau,\varvec{\mu}}} - \frac{{\tau \varvec{B}}}{K}\sum\limits_{n = 1}^{KN} {{ \log }_{2} \left({1 + \frac{{A_{n}}}{{A_{n} + \frac{\tau}{{1{-}\tau}}D_{n}}}D_{n} \mu_{n}} \right)} \\ & s.t. \sum\limits_{n = 1}^{KN} {\mu_{n} + S = 1}, \hfill \\ &\quad 0 < \tau < 1, \hfill \\ &\quad \mu_{n} \ge 0, \hfill \\ &\quad S \ge 0 \hfill \\ \end{aligned}$$

The unconstrained augmented Lagrangian penalty function of (37) can be written as

38 $$P\left({\varvec{x},\xi,\delta} \right) = - \frac{{\tau \varvec{B}}}{K}\sum\limits_{n = 1}^{KN} {{ \log }_{2} \left({1 + \frac{{A_{n}}}{{A_{n} + \frac{\tau}{{1{-}\tau}}D_{n}}}D_{n} \mu_{n}} \right)} - \xi \left(\sum\limits_{n = 1}^{KN} {\mu_{n} +S - 1}\right)+\frac{1}{2}\delta \left(\sum\limits_{n = 1}^{KN} {\mu_{n} +S_{1} - 1}\right)^{2},$$

where $\varvec{x} = \left({\tau,\varvec{\mu},S} \right)$, $S$ is the positive slack variable, $\xi$ and $\delta$ denote the Lagrangian multipliers and the penalty parameter, respectively.

In the $k$th iteration, $\varvec{x}^{(k + 1)}$ can be updated as follows

39 $$\varvec{x}^{(k + 1)} = {\text{argmin}}P(\varvec{x}^{(k)},\xi^{(k)},\delta^{(k)}).$$

Then the Lagrange multipliers can be obtained by

40 $$\xi^{(k+1)} =\xi^{(k)} {-}\delta^{(k)} \left({\sum\limits_{n = 1}^{KN} {\mu_{n}^{(k+1)} +S^{(k+1)} - 1}} \right) .$$

And the penalty parameters can be updated by

41 $$\delta^{(k + 1)} = \left\{{\begin{array}{ll} {\delta^{(k)},} & \quad {{\text{if}}\;\left| {\sum\limits_{n = 1}^{KN} {\mu_{n}^{(k+1)} +S^{(k+1)} - 1}} \right| \le \frac{1}{4}\left| {\sum\limits_{n = 1}^{KN} {\mu_{n}^{(k)} +S^{(k)} - 1}} \right|} \hfill \\ {\hbox{max} \{10\delta^{(k)},k^{2} \},} &\quad {{\text{otherwise}}.} \hfill \\ \end{array}} \right. .$$

By defing the constraint violation function in (42), we present the main steps of ALPF algorithm for PDFSR as shown in Algorithm 4.

42 $$C^{(-)} (\varvec{x}) = \sum\limits_{n = 1}^{KN} {\mu_{n} +S - 1}.$$

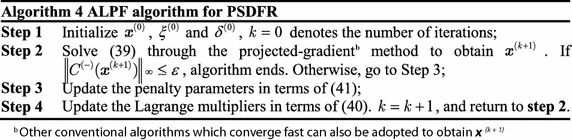


## Results and discussion

In this section, we shall first provide some numerical results to verify our theoretical analysis for the two proposed protocols and the optimization methods described in Section “Achievable Rate Optimization”. Then, the effects of various system parameters on the system achievable rate are also discussed, including the relay’s location, the number of antennas $N$ and the number of subcarriers $K$.

The distance between S and D is used to be the reference distance, which is denoted as $d_{\text{SD}}$, and the path loss factor is set to be 4. The variable $\phi \in (0,1)$ denotes the ratio of the distance between S and R, i.e. $d_{\text{SR}} =\phi d_{\text{SD}}$. Unless specifically stated, the available transmit power at S is set to be $\varvec{P}_{\text{S}} =30{\text{dBm}}$, and the total system bandwith is set to be $\varvec{B}= 1\,{\text{kHz}}$, so that each subcarrier is allocated with 1/*K* kHz. The total receiving noise at ${\text{R}}$ and ${\text{D}}$ over the total bandwidth is set to be $10^{- 6} {\text{W}}$, so that the noise over each subchannel is set to be $\sigma_{\text{R}}^{2} =\sigma_{\text{D}}^{2} =\frac{{10^{{{-}6}}}}{K}{\text{W}}$. We also assume that the energy conversion efficiency $\eta =1$, and such an assumption is also widely adopted in the exploration of system performance limit for the convenience of analysis (Xiong et al. 2015; Du et al. 2015; Nasir et al. 2013).

To show the performance gain of our optimized protocols, we also show the results of the non-optimized schemes as benchmarks. As for the non-optimized scheme of TSDFR, we apply the optimal sub-channel pairing, but $\alpha$ is set to be 0.10, $\mu_{n}$ and $\overline{\mu}_{{n^{\prime}}}$ are set as $\mu_{n} =\lambda_{1,n}/\sum\nolimits_{n = 1}^{KN} {\lambda_{1,n}}$ and $\overline{\mu}_{{n^{\prime}}} =\lambda_{{1,n^{\prime}}}/\sum\nolimits_{{n^{\prime} = 1}}^{KN} {\lambda_{{1,n^{\prime}}}}$, which are proportional to the singular value of each sub-channel. As for the non-optimized scheme of PSDFR, the optimal sub-channel pairing is also adopted, $\mu_{n}$ is set as $\mu_{n} =\lambda_{1,n}/\sum\nolimits_{n = 1}^{KN} {\lambda_{1,n}}$, which is similar to TSDFR.

### Verification of the analytical results

**Fig. 3:**
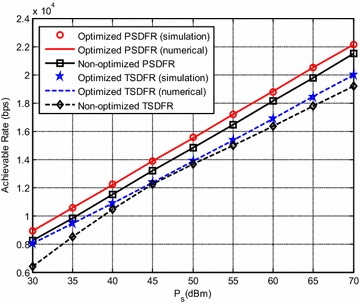
System achievable rate: numerical versus simulation with $K =N = 2$

Figure 3 verifies our theoretical analysis and the proposed ALPF algorithm of TSDFR and PSDFR. In the simulation, $K =N = 2$, $\phi$ is set to be 0.3 which means that the relay is located closer to the source. The numerical results are obtained by the optimization methods described in Section “Achievable Rate Optimization”, and the simulation results are obtained by computer searching.

**Fig. 4:**
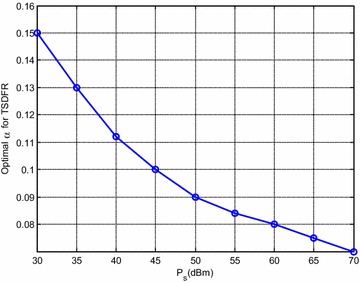
Optimal $\alpha$ for the optimized TSDFR

It can be observed from Fig. 3 that, firstly, for different available power $\varvec{P}_{\text{S}}$, the simulation results closely match with the numerical results which verifies our theoretical analysis and the proposed ALPF algorithm. Moreover, PSDFR achieves higher achievable rate than TSDFR. This may be due to the reason as follows: the performance of both TSDFR and PSDFR mainly depends on two factors: the *energy transfer pattern* and the *power allocation mode*. The energy transfer pattern affects the available power at R, which indirectly influences the information transfer on hop-2. Power allocation modes affect PSDFR and TSDFR on the available energy on each sub-channel, which also affects the information transfer essentially. From the protocols we can see that, in TSDFR, the time assignment factor $\alpha$ which affects the harvested energy over all subchannels is the same, whereas in PSDFR, each subchannel has its own factor $\rho_{i}$ ($i = 1,2,\ldots,KN$) to adjust the ratio between the energy harvesting and information transfer. That is to say, PSDFR can provide more flexibility to configure the system resources compared with TSDFR, which makes it achieve higher system performance. It also can be seen in Fig. 3 that when $\varvec{P}_{\text{S}}$ is around 45 dBm, the performance of the non-optimized TSDFR is very close to that of the optimized one. The reason is due to the reason that, when $\varvec{P}_{\text{S}}$ = 45 dBm, the optimal time assignment factor *α*^#^ = 0.10 (see Fig. 4), and in this section, we set $\alpha =0.10$ for the non-optimized TSDFR, which is exactly the same with the optimal $\alpha$. Therefore, when $\varvec{P}_{\text{S}}$ is around 45 dBm, it makes the performance of the non-optimized TSDFR very close to that of the optimized one.

Figure 4 shows the optimal $\alpha$ for the optimized TSDFR. It can be seen that, as $\varvec{P}_{\text{S}}$ at S increases, the optimal $\alpha$ decreases, which is due to the reason that, as the available power at S increases, enough harvested energy can be obtained at R, thus less $\alpha$ is needed.

**Fig. 5:**
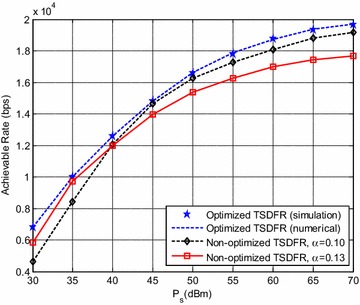
System achievable rate for optimal $\alpha$ of different $\varvec{P}_{\text{S}}$

In Fig. 5, we compare the performance of non-optimized TSDFR when $\alpha$ is set to be different. When $\alpha$ is set to be 0.13 which is exactly equal to the optimal $\alpha$ for $\varvec{P}_{\text{S}} =35\, {\text{dBm}}$, it can be observed that the performance of the non-optimized TSDFR is not close to the optimized TSDFR when $\varvec{P}_{\text{S}}$ is around 45 dBm, but is very close to the optimized one when $\varvec{P}_{\text{S}}$ is around 35 dBm. This is because that, $\alpha$ is set to be the optimal value of $\varvec{P}_{\text{S}} =35\,{\text{dBm}}$. Because both the optimized and non-optimized TSDFR apply optimal sub-channel pairing, and $\mu_{n}$ and $\overline{\mu}_{{n^{\prime}}}$ of two non-optimized TSDFR schemes are set as $\mu_{n} =\lambda_{1,n}/\sum\nolimits_{n = 1}^{KN} {\lambda_{1,n}}$ and $\overline{\mu}_{{n^{\prime}}} =\lambda_{{1,n^{\prime}}}/ \sum\nolimits_{{n^{\prime} = 1}}^{KN} {\lambda_{{1,n^{\prime}}}}$, there are reasons to believe that, $\alpha$ has greater impact on system achievable rate than power allocating factors.

### System performance versus ϕ

In this subsection, the effects of relay location on the system performance will be discussed.

**Fig. 6:**
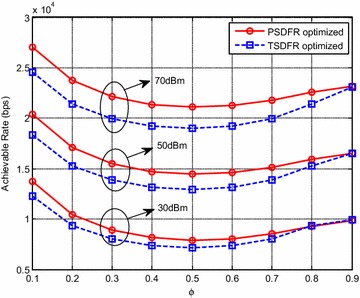
System achievable rate versus $\phi$, $K =N = 2$

As shown in Fig. 6, $\phi$ affects the system achievable rates of both PSDFR and TSDFR.

Specifically, as $\phi$ increases, the achievable rates first decrease and then increase, and achieve the minimum when the relay is deployed in the middle of S and D. The reason for such a phenomenon may be that, when R is closer to S, i.e., $\phi$ is small, higher energy harvesting efficiency could be achieved and more energy could be harvested at R, which makes the system achievable rate high. When R is closer to D, i.e., $\phi$ is large, better channel quality of R–D link is achieved, which could compensate for the loss brought by less harvested energy at R. Moreover, PSDFR achieves higher achievable rates than TSDFR, but when $\phi$ is large, the performance of TSDFR is close to that of PSDFR.

**Fig. 7:**
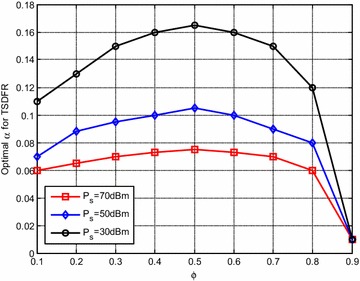
Optimal $$\alpha$$ versus $$\phi$$

Figure 7 shows the optimal $\alpha$ versus $\phi$. It can be observed that, as $\phi$ increases, the optimal $\alpha$ first increases and then decreases, which is to say that, when ${\text{R}}$ is in the middle of ${\text{S}}$ and ${\text{D}}$, $\alpha$ is relatively high. This is due to the fact that, when ${\text{R}}$ is far away from ${\text{S}}$, the energy harvesting efficiency becomes lower, ${\text{R}}$ needs higher $\alpha$ to collect enough energy to decode the information from ${\text{S}}$. And when ${\text{R}}$ is close to ${\text{D}}$, the channel quality of R–D link gets better, ${\text{R}}$ needs less energy to relay the information for ${\text{D}}$, which makes $\alpha$ get lower. It also can be seen that, for the same $\phi$, $\alpha$ gets lower when $\varvec{P}_{\text{S}}$ increases.

### System Performance vs the Number of Antennas $\varvec{N}$

**Fig. 8:**
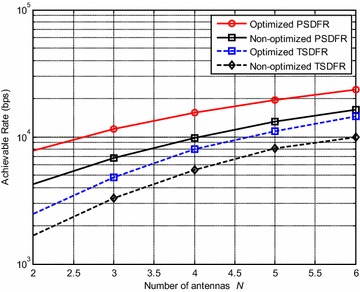
System achievable rate versus the number of antennas *N* with $K = 2$ subcarriers

Figure 8 shows the effect of the number of antennas $N$ on the system achievable rate. In the simulations, $K$ is set to be 2, and $N$ increases from 2 to 6. The results are averaged over 100 simulations. It can be observed that the system achievable rate increases as the number of antennas increases. This is due to the fact that more antennas yield more spatial subchannels, thus higher multiplex gain over subchannels can be achieved. Moreover, PSDFR can achieve higher achievable rate than TSDFR.

### System performance versus the number of subcarriers *K*

**Fig. 9:**
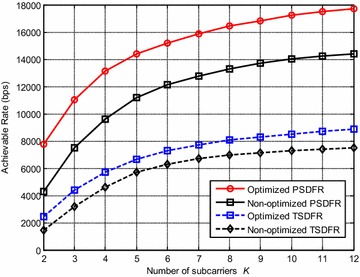
System achievable rate vs the number of subcarriers *K* with $N = 2$ antennas

In this subsection, the system performance vs the number of subcarriers $K$ will be discussed. In the simulations, $N$ is set to be 2, and $K$ increases from 2 to 12, the results are averaged over 100 simulations. It can be observed from Fig. 9 that the system achievable rates of both schemes increase as the number of subcarrier increases, and PSDFR achieves higher achievable rate than TSDFR, but the increasing rate of both curves become slower with the increment of $K$. This is due to the fact that, more subcarriers yields more subchannels, which could produce more flexible system configuration to increase system achievable rate. However, with a fixed system total bandwidth, more subcarriers results in smaller bandwidth allocated to each subcarrier. Thus increasing $K$ can not always increase system achievable rate, but there exists a trade-off.

### System performance versus *τ*

In this subsection, the effects of relay location on the parameter $\tau$ which describes the time division pattern between the two phases will be discussed. The numerical results are obtained by the optimization methods described in Section “Achievable Rate Optimization”, and the simulation results are obtained by computer searching.

**Fig. 10:**
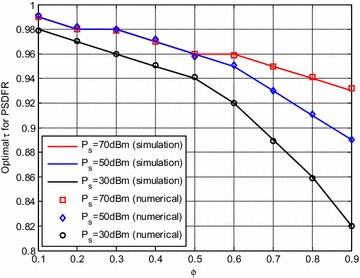
Optimal $$\tau$$ versus $$\phi$$

It can be observed from Fig. 10 that, the simulation results closely match with the numerical results, and the optimal $\tau$ decreases as $\phi$ increases. That is to say, S needs more time to transfer power and information to get higher system achievable rates when R is close to S. This may be due to the reason that, when R is close to S, the channel quality of R–D link gets worse, to guarantee that R collect enough energy to successfully transmit signal to D, S needs more operating time to ensure the energy harvesting at R.

It is also shown in Fig. 10 that when S increases the transmit power, it’s better for it to spend more time in Phase 1 to achieve better system performance. We can see that, the optimal $\tau$ for all cases is above 0.8, obviously, the common division of the total transmission time into two equal phases in previous work applying PS-based receiver is not optimal. And the attenuation caused by the path loss due to the long distance is still an important influence factor of energy harvesting’s performance.

## Conclusion

This paper investigated the system achievable rate and optimization for the MIMO–OFDM DF relaying system with an EH relay. Firstly, we proposed two protocols, TSDFR and a flexible PSDFR protocol to enable the simultaneous information processing and energy harvesting at the relay. In order to explore the system performance limit, we formulated two optimization problems to maximize the system achievable rate. Based on some explicit theoretical results of the optimization problems, we designed an ALPF algorithm for them. Numerical results were provided to verify our analytical results and the effectiveness of the proposed ALPF algorithm.

It is shown that, the optimized results of the proposed TSDFR and PSDFR protocols provide impressive performance gain compared with the non-optimized schemes. Moreover, PSDFR outperforms TSDFR to achieve higher achievable rate. It is also shown that the relay position greatly affects the system performance, and relatively worse achievable rates are achieved when the relay is placed in the middle of the source and the destination. This is different from the MIMO–OFDM DF relaying system without SWIPT. Besides, increasing the number of system antennas or subcarriers can both improve the system performance, but with a fixed system total bandwidth, there exists a trade-off between the number of system subcarriers and the achievable rates.

In addition, the optimal factor which indicates the time division pattern between the two phases in the PSDFR protocol is always above 0.8, which means that, the common division of the total transmission time into two equal phases in previous work applying PS-based receiver is not optimal. Besides, we find some differences between AF and DF systems. For example, in the AF system of Reference (Xiong et al. 2015), as the relay node moves from the source to the destination, the optimal time assignment factor in TS-based protocol will decrease to achieve higher system performance. But in DF systems, as the relay node moves from the source to the destination, the optimal time assignment factor first increases and then decreases, which is to say that, when the relay is in the middle of the source and the destination, the optimal time assignment factor is relatively high.

## Abbreviations

AF: amplify-and-forward
ALPF: augmented Lagrangian penalty function
DF: decode-and-forward
EH: energy harvesting
MIMO: multiple-input multiple-output
OFDM: orthogonal frequency division multiplexing
RF: radio-frequency
SNR: signal-to-noise ratio
SWIPT: simultaneous wireless information and power transfer
TSDFR: time switching-based decode-and-forward relaying
PSDFR: power splitting-based DF relaying

## Footnotes

1 For the detailed architectures of the two practical relay receivers, please refer to (Zhou et al. 2013), and for the detailed process in which how do the time switching and power splitting schemes work for EH relaying systems, please refer to (Du et al. 2015; Nasir et al. 2013).

2 In this phase, only energy is transferred, and all the energy is carried on the vector.

3 To see the detailed solution and proof, please reffer to (Xiong et al. 2015).
